# Special Issue “Advances in Friction, Wear-Resistant and Solid-Lubricating Properties of Materials”

**DOI:** 10.3390/ma19122588

**Published:** 2026-06-16

**Authors:** Xiangli Wen

**Affiliations:** Institute of Technology for Carbon Neutralization, Interdisciplinary Research Center for Advanced Energy, Yangzhou University, Yangzhou 225127, China; xlwen@yzu.edu.cn

## 1. Introduction and Scope

On behalf of the editorial team, it is my great pleasure to introduce this Special Issue (SI) of Materials titled “*Advances in Friction, Wear-Resistant and Solid-Lubricating Properties of Materials*.” As the Guest Editor, I am honored to present a curated collection of nine high-quality research articles and reviews that collectively address some of the most pressing challenges and innovative breakthroughs in the field of modern tribology.

Friction, wear, and lubrication are fundamental phenomena that dictate the efficiency, reliability, and lifespan of countless mechanical systems, from precision instruments to heavy industrial machinery and aerospace components. As global industries pivot towards higher efficiency, sustainability, and extreme operating conditions (such as higher temperatures, heavier loads, and corrosive environments), the demand for advanced materials and innovative lubrication strategies has never been greater. This Special Issue was conceived to provide a platform for cutting-edge research that pushes the boundaries of material performance, tribological understanding, and computational modeling.

The nine articles featured in this Special Issue represent a diverse and comprehensive spectrum of tribological research. They are broadly categorized into three interconnected domains: (1) advanced manufacturing and surface engineering for enhanced wear resistance; (2) innovations in lubricants and solid-lubricating additives; (3) tribological behavior and failure mechanisms under specific service conditions. Together, they paint a vivid picture of the current state and future directions of materials tribology. We will provide detailed introductions to six of the three parts of the literature.

## 2. Overview of Published Articles

### 2.1. Advanced Manufacturing and Surface Engineering for Enhanced Wear Resistance

The relentless pursuit of extended component lifetimes and superior mechanical performance has positioned advanced manufacturing and surface engineering at the forefront of tribological research. Modifying surface properties or employing novel additive manufacturing techniques allows engineers to tailor materials specifically for harsh operating environments. Three papers in this Special Issue exemplify these cutting-edge approaches.

First, Fu et al. (Contribution 1) present a groundbreaking study that combines materials science with artificial intelligence to optimize laser cladding processes. Recognizing the complexity of balancing alloy composition and processing parameters, the authors constructed a comprehensive database linking these variables to the wear properties of Ni-based self-fluxing alloy (SFA) coatings. By employing Pearson’s correlation coefficient and Random Forest (RF) feature importance assessments, they identified carbon (C) and boron (B) as the most influential elements. The integration of an RF model with a Genetic Algorithm (GA) yielded a highly efficient collaborative optimization system. Remarkably, the optimized coating achieved an average friction coefficient of merely 0.34, alongside significantly enhanced solid solution strengthening (110 MPa) and a high hard-phase content (52%) [1]. This study not only provides a superior wear-resistant material but also establishes a powerful data-driven methodological framework for optimizing laser cladding and other advanced manufacturing processes.

Second, Wu et al. (Contribution 2) address the inherent poor wear and corrosion resistance of lightweight magnesium alloys, which often hinders their broader industrial adoption. Focusing on the ZE52 magnesium–zinc–rare earth alloy, the authors utilized the DC magnetron sputtering process to deposit CrSiN films, preceded by a CrSi buffer layer to ensure robust adhesion. By systematically modulating the substrate bias voltage, they discovered that a nanocomposite structure—comprising CrN nanocry stallites embedded in an amorphous Si_3_N_4_ matrix—yielded optimal mechanical properties. Specifically, at a bias voltage of −50 V and a substrate temperature of 250 °C, the coated alloy exhibited a peak hardness of 16.5 GPa and a Young’s modulus of 187.4 GPa. Consequently, this specific coating demonstrated the lowest wear rate (2.2 × 10^−6^ mm^3^·m^−1^·N^−1^) under a 4 N load, alongside exceptional corrosion resistance in a 3.5% NaCl solution. This work elegantly demonstrates how precise control over thin-film deposition parameters can unlock the industrial potential of lightweight structural materials.

Third, Yasa et al. (Contribution 4) explore the intricacies of Additive Friction Stir Deposition (AFSD) of Al6061 aluminum alloy, a solid-state additive manufacturing method. The quality and mechanical integrity of additively manufactured parts are heavily dependent on process stability. The authors implemented a sophisticated in situ process monitoring setup, incorporating sensors to track temperature, forces, vibrations, and sound. By comparing the process with and without a Proportional–Integral–Derivative (PID) controller, they demonstrated the critical role of thermal management. Their findings revealed substantial thermal gradients and documented the fluctuations in temperature and force during the initial layers of deposition until stability was achieved. Furthermore, the study linked these process dynamics to the final residual stress distribution within the part, identifying compressive stresses at the core and tensile stresses in the outer regions. This research underscores the vital link between manufacturing process control, material properties, and the resulting mechanical performance.

### 2.2. Failure Mechanisms Under Specific Tribological Conditions

While advanced materials can withstand extreme conditions, the role of lubricants and solid-lubricating additives remains indispensable in minimizing friction and preventing adhesive wear. Two contributions in this Special Issue delve into the nuanced chemistry and physics of lubrication, exploring both macroscopic grease formulations and molecular-level tribofilm formation.

Senyk et al. (Contribution 5) investigated the tribological performance of hexagonal boron nitride (h-BN) as a solid lubricant additive in both lithium and calcium greases. h-BN is renowned for its excellent thermal stability and lubricious properties, yet its performance is highly dependent on particle size, concentration, and the base grease matrix. Through rigorous ball-on-flat reciprocating tests under varying loads, the researchers found that greases formulated with 3% h-BN—specifically those with an average particle size of 130 nm and a high nanoparticle content—yielded the most favorable results. Compared to the base greases, this formulation reduced friction by up to 9.7% and wear by up to 69.2% in lithium grease, and reduced friction by up to 18.2% and wear by up to 70.2% in calcium grease. Scanning Electron Microscopy (SEM) and Energy Dispersive X-ray Spectroscopy (EDS) analyses revealed that the dominant lubrication mechanisms were the shearing-sliding effect and the surface-mending effect, where the h-BN particles effectively repaired and protected the contacting surfaces. This study highlights the critical importance of matching the additive characteristics to the base lubricant matrix to maximize tribological benefits.

Complementing the macroscopic approach to grease, Wen et al. (Contribution 9) provided a profound mechanistic insight into synergistic lubrication at the nanoscale. Focusing on ethylene glycol-based hydraulic fluids (HFC), which are prized for their fire-retardant properties, the authors explored the combination of TM-104 and Vanlube 672 additives. They discovered a remarkable dual-additive synergy that facilitates the in situ formation of a nanostructured bilayer tribofilm at the friction interface. The optimized formulation (0.5 wt.% TM-104 and 2.0 wt.% Vanlube 672) consisted of a 6 nm thick inner layer rich in P_x_O_y_ and a 140 nm thick outer layer of amorphous carbon [2]. This autonomously assembled, structurally graded tribofilm delivered exceptional performance: a 31% improvement in lubricity, a 71% increase in wear resistance, and an astounding 577% enhancement in extreme-pressure load capacity. The inner P_x_O_y_ layer provided strong chemisorption bonds with the substrate, while the outer carbon layer dissipated energy through shear-induced graphitization. This pioneering work establishes a new paradigm for designing multi-functional lubricant additives capable of operating under extreme pressures.

## 3. Future Perspectives in Tribology: Trends and Trajectories

While the studies compiled in this SI offer compelling solutions to contemporary tribological problems, they also point toward transformative trends that will define the future of the field. Drawing upon broader advancements in materials science and engineering, three key trajectories stand out: the integration of artificial intelligence, the discovery of novel two-dimensional (2D) solid lubricants, and the push toward sustainability and extreme-environment resilience.

### 3.1. The Rise of AI and Physics-Informed Machine Learning

The success of data-driven models, such as the one employed by Fu et al. [1], signals a permanent shift in how tribological systems are designed and understood. Moving forward, the field is rapidly progressing from simple empirical correlations to sophisticated Physics-Informed Neural Networks (PINNs) [3,4]. Future tribological models will seamlessly integrate multiphysics phenomena—such as heat transfer, fluid–structure interaction, and microstructural evolution—into cohesive computational frameworks. This will allow researchers to predict friction and wear regimes with unprecedented accuracy, moving away from the traditional “trial-and-error” experimental approaches. Ultimately, this synergy between machine learning and tribology will enable the accelerated discovery of new materials, where algorithms suggest optimal alloying elements or coating architectures tailored for specific industrial applications [4,5].

### 3.2. Next-Generation 2D Solid Lubricants

For decades, materials like MoS_2_ have dominated the landscape of solid lubrication. However, their sensitivity to environmental conditions, such as humidity and temperature, limits their universal applicability. The future of solid lubrication lies in the exploration of novel 2D nanomaterials. Among these, MXenes have recently emerged as a groundbreaking solution, offering exceptional lubrication performance at micro- and nano-scales [6]. Their unique combination of mechanical robustness, ultra-low shear resistance, and tunable surface chemistry allows them to form conformal, atomically thin lubricating films that outperform conventional solid lubricants in demanding environments [7]. Future research will undoubtedly focus on overcoming current challenges related to the oxidation stability and large-scale synthesis of these materials, paving the way for their integration into next-generation protective tribological coatings [6,7].

### 3.3. Tribology for a Sustainable and Hydrogen-Powered Future

As global industries strive to meet stringent carbon neutrality goals, tribology is evolving beyond mere component longevity to encompass systemic energy efficiency and environmental stewardship. This is particularly evident in the rapid development of the hydrogen economy. As highlighted by Krella [8], understanding material degradation in hydrogen-rich environments is paramount. Future tribological research must develop innovative surface engineering strategies and advanced ceramic or diamond-like carbon (DLC) coatings that can simultaneously withstand hydrogen embrittlement, extreme contact pressures, and cryogenic or high-temperature conditions inherent to hydrogen fuel cells and advanced energy storage systems [8,9,10,11,12].

## 4. Conclusions

The nine papers compiled in this Special Issue offer a compelling snapshot of ongoing research in the friction, wear, and lubrication of advanced materials. From atomic-level insights into tribofilm growth and hydrogen diffusion to macroscale optimizations of industrial greases and thick-section steels, the breadth of research is remarkable. Crucially, these works collectively underscore that the future of tribology is inherently interdisciplinary.

## Data Availability

No new data were created or analyzed in this study. Data sharing is not applicable to this article.
